# Consistent condom use and its correlates among female sex workers at hair salons: a cross-sectional study in Zhejiang province, China

**DOI:** 10.1186/s12889-017-4891-6

**Published:** 2017-11-28

**Authors:** Qiaoqin Ma, Jianmin Jiang, Xiaohong Pan, Gaofen Cai, Hui Wang, Xin Zhou, Tingting Jiang, Lin Chen

**Affiliations:** Zhejiang Provincial Center for Disease Control and Prevention, No.3399, Binsheng Road, Hangzhou, 310051 China

**Keywords:** Condom, Correlate, Female sex worker, Hair salon, China

## Abstract

**Background:**

This study investigated condom use among female sex workers (FSWs) at hair salons during commercial sexual interactions over 1 month. We explored the associations of such use with various sexual behaviours, HIV/STI risk perception and related knowledge, self-efficacy regarding condom use, exposure to behavioural interventions, and other factors. This type of information has not been reported in China and is critical for designing and modifying programs aimed at preventing HIV/STI transmission in this group of FSWs and their clients.

**Methods:**

Our data were derived from a large cross-sectional study conducted among low-tier FSWs in 21 counties within Zhejiang province, China. Data were collected from September to November 2013.Bivariable and multiple logistic regression analyses were used to identify factors associated with self-reported consistent condom use among FSWs working at hair salons.

**Results:**

Of 1682 FSWs working at hair salons, 50.5% consistently used condom with clients during the previous month. Multivariable analysis revealed that condom use for commercial sex, consistent vaginal douching after commercial sex, adopting contraceptive measures, high scores on perceived self-efficacy regarding condom use, and exposure to interventions were associated with self-reported consistent condom use; early initiation of commercial sex, experiences with oral sex, rarely/sometimes/often using oral contraceptives, and having seen a doctor were associated with not using condoms.

**Conclusions:**

Commercial sex is not effectively protected, and behavioural interventions targeting FSWs should take into account the various factors that are correlated to condom use.

## Background

Heterosexual commercial sex has played an important role in the transmission of HIV and other sexually transmitted infections (STIs). A meta-analysis estimated that, due to biological, behavioural, and structural risk factors, female sex workers (FSWs) are 13.5 times more likely to be living with HIV than other women of similar reproductive age in low- and middle-income countries [1]. Globally, 15% of female HIV infections in 2011 have been attributed to sex workers [2].

FSWs work in different venues and can be classified as high-, middle-, and low-tier based on the venues where they operate [3]. The sex-work tiers usually represent the socioeconomic level, the sex-selling environment, the type of client, and the risky behaviours of FSWs in China [4]. Low-tier refers to FSWs who generally work on the streets and at small venues, such as hair salons, massage parlours, roadside shops, and so on, and charge much less for each sex act [5].

Compared with high-tier FSWs, low-tier FSWs are generally older, less educated, and more likely to be married or separated/divorced/widowed [6, 7]. These sociodemographic characteristics, which reflect greater vulnerability including poorer attraction to clients, lower socio-economic level, may be associated with higher observed rates of condomless sex [6] and lower use of HIV prevention services [6, 8]. Studies have shown that FSWs working in low-tier venues tend to have more clients but use condoms infrequently because they are offered extra payment for condomless sex [9–11], they fear losing clients [12], and they spend very little money on healthcare, which hinders their access to STI diagnosis and treatment [13]. Men who patronise low-tier FSWs in China tend to be equally uneducated and of low socioeconomic status [5, 14, 15]. They may also be at a higher risk of HIV infection as low-tier FSWs are usually less likely to practice safer sex compared with their counterparts working at high-tier venues [14]. In contrast, men who visit high-tier FSWs usually have a high socioeconomic status, and they may be more concerned about their health and more likely to practice protected sex [14, 16].

Previous studies have found that low-tier FSWs in China face a high risk of HIV infection [17] and STIs [13, 18]. Low-tier FSWs in China are more likely to experience a higher prevalence of HIV infection and STIs, such as syphilis, HSV-2, and gonorrhoea, than their high-tier counterparts [15, 19]. The HIV prevalences among FSWs from high-, middle- and low-tier venues nationwide have been found to differ substantially, at 0.59%, 0.92%, and 1.10%, respectively [20]. HIV infections were significantly more frequent among FSWs working in higher-risk venues as opposed to in other venues (17.9% vs. 5.7%) [17]. Low-tier FSWs experienced the highest prevalence of syphilis (9.7%); this was followed by middle-tier (4.3%) and high-tier (2.2%) FSWs [21]. A systematic review reported that low-tier FSWs are about twice as likely to have syphilis compared with higher-tier ones [7].

Evidence concerning the higher prevalence of HIV and STIs among low-tier FSWs in China indicates insufficient condom use and higher risks of contracting HIV/STI for this group of FSWs. In 2013, we conducted a large study to investigate the characteristics of sexual behaviours and other HIV/STI-related factors in low-tier FSWs in Zhejiang province, China. In this study, we categorised low-tier FSWs as those who work on streets and in small venues with fewer than nine FSWs. Of these small venues, hair salons were very common and popular places for clients to seek sex services in China. These establishments appear to be hair salons with regard to both interior and exterior decor, but they usually provide only sexual services and do not offer haircutting services.

Some of the extant literature on condom use among low-tier FSWs has focussed on sociodemographic characteristics, HIV-related knowledge, exposure to interventions, HIV testing, and factors related to the sex trade, including number of clients, trading price [22–25], condom use, self-efficacy [26], and so on. However, there has been no research attention paid to FSWs who work at hair salons and no data derived from multiple work environments; furthermore, existing studies have not explored many relevant behaviours, such as oral sex, anal sex, contraception, and vaginal douching. Thus, it is important to understand condom use and related factors to guide the design and modification of programs targeting this group of FSWs to prevent HIV/STI transmission. The aim of this study was to investigate condom use for commercial sex purposes within this group and to examine associated behaviours, use of other contraceptives, STI symptoms, doctors’ visits, perceptions of HIV/STI, HIV/STI-related knowledge, self-efficacy regarding condom use, and exposure to HIV-related interventions.

## Methods

### Location

**Fig. 1:**
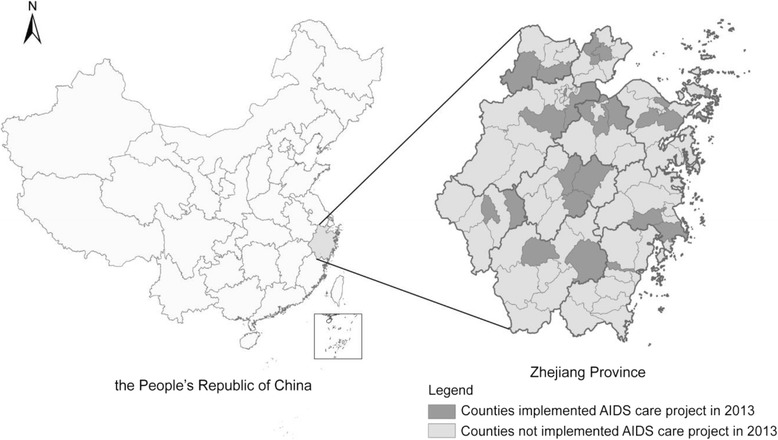
The location of Zhejiang province in China and the location of counties which implemented and not implemented AIDS care project in Zhejiang province, 2013

The participants in this study were drawn from a large cross-sectional study conducted in 21 counties of Zhejiang Province from September to November 2013. Zhejiang province covers an area of 105.5 thousand square kilometres, is located along the east coast of China, and has a population of 55.4 million people [27]. The province includes 90 counties in 11 prefectures [27]. Of these, 22 counties implemented the AIDS Care project in 2013. The AIDS Care project was a pilot program initiated by the national Ministry of Health and the Provincial Bureau of Health to support local areas in HIV prevention practices and policies. The program also promoted HIV prevention measures, including HIV-related education, behavioural interventions, counselling and screening, antiretroviral treatments, and so on. Of these 22 counties, one did not participate in our study because no low-tier FSWs were identified; the remaining 21 counties were distributed across all 11 prefectures of Zhejiang province (see Fig. 1).

### Participants and recruitment

Women met the inclusion criteria for participation if they were currently engaging in sex work on the street and or at small venues, including hairs salons, massage shops, roadside shops, and other venues with fewer than nine FSWs. All the women who worked in these venues were research participants, and all the counties participating in this study conducted a survey to confirm the location of low-tier FSWs in their area and then developed a plan to complete this study.

In total, 2648 low-tier FSWs participated in the study: 1687 worked at hair salons, 277 worked at roadside shops, 413 worked on the street, and 271 worked at other locations. Of the 1687 FSWs who worked at hair salons, 1682 responded to the following question included in our study: “How frequently did you use condoms with your clients over the last month?”, and were included in the analysis of this manuscript. The possible response options for this question include never, rarely, sometimes, often, or consistently use condom.

All participants who met the criteria for recruitment were informed of the study’s purpose and assured that their privacy and confidentiality would be firmly protected. Every participant was invited to voluntarily participate in the study. The interview was conducted in a private and quiet space within the venues. The questionnaires were anonymous, and participants were interviewed by the staff of local Centers for Disease Control and Prevention (CDCs), who were trained by research teams prior to the study.

### Questionnaire development

The questionnaire used in this study was based on instruments used for HIV sentinel surveillance among FSWs and reviews of domestic and foreign literature regarding low-tier FSWs; it was then revised again through repeated discussions with the research team and consultation with the staff of the CDCs who were responsible for behavioural interventions with FSWs in the counties studied. Finally, the questionnaire was modified through two pilot surveys with low-tier FSWs in two counties.

### Measures

Self-reported consistent condom use with commercial clients during the most recent month was used as a dependent variable in the analysis. The independent variables included sociodemographic characteristics, factors related to sexual behaviour and HIV/STI risk perception, and scales measuring HIV/STI-related knowledge, self-efficacy regarding condom use, and interventions experienced. We computed Cronbach’s alpha coefficients for the internal consistency of each scale and the ranges of scores; participants were categorised into three groups with different levels of scores based on the frequency distribution of each scale.

The HIV/STI-related knowledge scale included six statements about whether HIV cases acquired through sexual intercourse were increasing, whether HIV/STIs could be transmitted through oral sex, whether STIs make a person more vulnerable to HIV, whether correct condom use could effectively reduce the transmission of HIV/STIs, whether a person infected with HIV may lack visible symptoms, and whether STI infections may be detectable but asymptomatic. The following three response options to these six statements were available: “correct,” “incorrect,” and “unsure.” The scores for this scale ranged from 0 to 6, with 6 reflecting a high level of knowledge, 5 reflecting a middle level of knowledge, and 0–4 reflecting a low level of knowledge. The Cronbach’ s alpha coefficient for this scale was 0.812.

The scale measuring self-efficacy regarding condom use consisted of three statements addressing whether a participant could persuade a client to use a condom when a client had refused to do so during a previous sexual encounter, whether she could refuse sex when a client refused to use a condom, and whether she could insist on using a condom with clients every time. The possible responses were “I can,” “I can’t,” and “I’m not sure.” The scores for this scale ranged from 0 to 3, with 3 reflecting a high level of self-efficacy, 1–2 reflecting a middle level of self-efficacy, and 0 reflecting a low level of self-efficacy. The Cronbach’s alpha coefficient for this scale was 0.923.

The scale measuring experience with HIV-related interventions addressed the interventional services to which a participant was exposed during the most recent 6 months. It included four statements addressing whether a participant ever received any educational material, condoms, face-to-face education from medical staff, peer education, or other services. This scale had a Cronbach’s alpha coefficient of 0.619, and its scores ranged from 0 to 4, with scores of 3–4 classified as having received a high level of intervention, 0–1 classified as having received no or one type of intervention.

### Statistical analysis

Data were analysed using SPSS for Windows (Version 17.0; SPSS Inc., Chicago, IL, USA). The prevalence of self-reported consistent condom use and the frequency distributions of the independent variables were determined using bivariable analysis. The association between the dependent variable and each independent variable was computed using an odds ratio (OR) with a corresponding 95% confidence interval (95% CI) and a *P*-value based on a chi-square test of proportions. Variables identified as significantly associated with self-reported consistent condom use in the bivariable analyses (all being shown in Table 1, Table 3, and Table 4) were then entered into a logistic regression model to determine the independent contribution of each factor to predicting self-reported consistent condom use. Chi-square analysis was used to compare the difference between those who performed oral sex and those who did not. A *P*-value of less than 0.05 was considered statistically significant for these bivariable and multivariable analyses.

**Table 1:** Demographic characteristics of female sex workers working at hair salons (*n* = 1682)

Variable	N	%
Age^a^		
≤ 25	590	35.1
26–30	388	23.1
≥ 31	697	41.4
Residence^a^		
Local area	189	11.2
Other area in this province	230	13.7
Other province	1263	75.1
Education		
Primary school and below	559	33.2
Junior high school	940	55.9
High school and above	179	10.6
Marital status		
Unmarried	538	32.0
Married	940	55.9
Cohabiting	65	3.9
Widowed/divorced	139	8.3
Children		
No	659	39.1
Yes	974	57.9
Income per month		
< 3000	476	28.3
3000–4000	697	41.4
> 4000	447	26.6

^a^This variable was significantly associated with self-reported consistent condom use in bivariable analysis

## Results

### Sociodemographic characteristics

Of the 1682 participants, 35.1% were aged 25 years or younger, and 41.4% were older than 30 years of age (Table 1). Overall, 75.1% came from provinces other than Zhejiang. In terms of education, 33.2% had received, at most, primary school education, and 10.6% had received at least high school education. Overall, 32% were not married and 55.9% were married; 57.9% had at least one son or daughter. In terms of financial status, 28.3% earned an income of less than 3000 Yuan for 1 month, and 26.6% earned an income of over 4000 Yuan (one Yuan is equal to about 0.15 US dollar).

Bivariable analysis indicated that educational level, marital status, having a son/daughter, and incomes were not associated with self-reported consistent condom use. The FSWs from provinces other than Zhejiang compared with those from the local area (OR = 0.70, 95% CI = 0.52–0.95), the FSWs who were aged 26–30 years (OR = 0.72, 95% CI = 0.56–0.94) and over or equal to 31 years old (OR = 0.63, 95% CI = 0.51–0.79) compared with those under the age of 26 years old, were negatively associated with condom use.

### Condom use

In total, 50.5% of the participants always used condoms with clients over the previous month. The proportions of participants who never, rarely, sometimes, and often used condoms over this same period were 1.6%, 11.8%, 14.6%, and 21.5%, respectively (Table 2). Of the 833 participants who did not always use condoms with clients, 83.2% attributed this to the fact that their clients were unwilling to use them. The other reasons listed included attracting clients, the reduction of pleasure, not believing they were necessary, not having one readily available, and other reasons: 22.0%, 9.6%, 6.8%, 5.2%, and 12.1%, respectively.

**Table 2:** Condom use with clients and reasons for inconsistent use

Variable	N	%
Condom use with clients during the past month (*n* = 1682)		
Never	27	1.6
Rarely	199	11.8
Sometimes	245	14.6
Often	362	21.5
Always	849	50.5
Reason for inconsistent use with clients (*n* = 833)^a^		
Client unwilling	693	83.2
To attract clients	183	22
To reduce the duration sex	80	9.6
Unnecessary	57	6.8
Wanted to use but no condom available	43	5.2
Other	101	12.1

^a^The participants could give multiple responses

Three hundred sixty participants lived together with their spouses or boyfriends during this survey. The proportions of them who never, rarely, sometimes, often and consistently used condoms with their current spouses or boyfriends were 32.2%, 20.0%, 26.9%, 11.9%, and 8.9%, respectively.

### Correlates of self-reported consistent condom use during the previous month

#### Sexual behaviour correlates

Bivariable analysis indicated that the FSWs being negatively associated with condom use were those who initiated sex work at the age of 20–29 years (OR = 0.57, 95% CI = 0.42–0.76) or over 29 years (OR = 0.60, 95% CI = 0.43–0.83) versus those who initiated sex work before they were 20 years old; those who engaged in commercial sex in two provinces (OR = 0.58, 95% CI = 0.46–0.72) and those working in more than two provinces (OR = 0.33, 95% CI = 0.24–0.44) versus those engaging in commercial sex and working in one province; those who had engaged in commercial sex for more than 24 months versus those who had worked 1–12 months (OR = 0.60, 95% CI = 0.48–0.75); those who had experienced anal sex versus those who had not (OR = 0.45, 95% CI = 0.29–0.69); those who performed oral sex versus those who did not (OR = 0.52, 95% CI = 0.41–0.66); those who had shown STI symptoms during the previous 6 months (OR = 0.57, 95% CI = 0.42–0.76) versus those who had not; and those who rarely (OR = 0.25, 95% CI = 0.18–0.34), sometimes (OR = 0.18, 95% CI = 0.12–0.25), or often (OR = 0.18, 95% CI = 0.11–0.28) used oral contraceptives versus those who never used them (Table 3).

**Table 3:** Correlates of consistent condom use among female sex workers working at hair salons (*n* = 1682)

Variable	Total (N, %)	Consistent condom use (%)	Crude OR	*P* value
Age of first commercial sex act				
< 20	230 (13.7)	144 (62.6)	1	
20–29	1013 (60.2)	493 (48.7)	0.57 (0.42–0.76)	0
> 30	423 (25.1)	212 (50.1)	0.60 (0.43–0.83)	0.002
Condom use during first commercial sex act				
No	503 (29.9)	115 (22.9)	1	
Yes	1174 (69.8)	732 (62.4)	5.59 (4.40–7.10)	0.000
Province number for commercial sex				
1	879 (52.3)	519 (59.0)	1	
2	542 (32.2)	246 (45.4)	0.58 (0.46–0.72)	0.000
≥ 3	260 (15.5)	83 (31.9)	0.33 (0.24–0.44)	0.000
Tenure in commercial sex industry				
1–12 months	703 (41.8)	393 (55.9)	1	
13–24 months	408 (24.3)	208 (51.0)	0.82 (0.64–1.05)	0.113
> 24 months	568 (33.8)	246 (43.3)	0.60 (0.48–0.75)	0.000
Price for commercial sex during the previous month				
≤ 100	953 (56.7)	458 (48.1)	1	
> 100	723 (43.0)	387 (53.5)	1.25 (1.03–1.51)	0.027
Number of commercial sex partners during the previous month				
< 15	552 (32.8)	253 (45.8)	1	
15–29	512 (30.4)	297 (58.0)	1.63(1.28–2.08)	0.000
> 29	607 (36.1)	291 (47.9)	1.09 (0.86–1.37)	0.473
Anal sex during the previous month				
No	1583 (94.1)	817 (51.6)	1	
Yes	96 (5.7)	31 (32.3)	0.45 (0.29–0.69)	0.000
Oral sex during the previous month				
No	1323 (78.8)	714 (54.0)	1	
Yes	356 (21.2)	134 (37.6)	0.52 (0.41–0.66)	0.000
Douched vaginally after commercial sex during the previous month				
Never	142 (8.4)	38 (26.8)	1	
Rarely	206 (12.2)	62 (30.1)	1.18 (0.73–1.90)	0.499
Sometimes	206 (12.2)	68 (33.0)	1.35 (0.84–2.16)	0.214
Often	371 (22.1)	171 (46.1)	2.34 (1.53–3.58)	0.000
Always	756 (44.9)	510 (67.5)	5.67 (3.80–8.48)	0.000
STI symptoms during the previous 6 months				
No	1459 (86.7)	763 (52.3)	1	
Yes	214 (12.7)	82 (38.3)	0.57 (0.42–0.76)	0.000
Oral contraceptive use during the previous month				
Never	938 (55.8)	587 (62.6)	1	
Rarely	256 (15.2)	76 (29.7)	0.25 (0.18–0.34)	0.000
Sometimes	199 (11.8)	45 (22.6)	0.18 (0.12–0.25)	0.000
Often	114 (6.8)	26 (22.8)	0.18 (0.11–0.28)	0.000
Always	173 (10.3)	114 (65.9)	1.16 (0.82–1.63)	0.407
Current contraception				
No	752 (44.7)	340 (45.2)	1	
Yes	930 (55.3)	509 (54.7)	1.47 (1.21–1.78)	0.000

The FSWs more likely to use condoms were those who used condoms during their first commercial sex act versus those who did not (OR = 5.59, 95% CI = 4.40–7.10); those who charged an average price over 100 Yuan versus those who charged less than (OR = 1.25, 95% CI = 1.03–1.51); those who had experienced commercial sex with 15–29 partners versus those who had experienced commercial sex with fewer partners (OR = 1.63, 95% CI = 1.28–2.08); those who often (OR = 2.34, 95% CI = 1.53–3.58) or always (OR = 5.67, 95% CI = 3.80–8.48) vaginally washed after commercial sex versus those who never washed; and those who used contraception versus those who did not (OR = 1.47, 95% CI = 1.21–1.78). Contraception refers to intrauterine devices, tubal ligation, or the Norplant method; those FSWs who adopted one of these measures were considered to be using contraception.

Three hundred fifty six FSWs (21.2% of the participants) performed oral sex during the previous month. Those who performed oral sex were significantly more likely to be younger than 26 years old (43.5% vs. 32.7%, *P* < 0.001), to be unmarried (43.3% vs. 28.9%, P < 0.001), to have earned less than 3000 Yuan (38.5% vs. 25.6%, P < 0.001), to have had more than 14 sexual partners (83.1% vs. 63.0%, P < 0.001), to engage in anal sex (19.7% vs. 2.0%, P < 0.001), and to show STI-related symptoms (21.6% vs. 10.3%, P < 0.001) than those who did not.

#### Correlates of preventive behaviour and awareness

Those who had seen a doctor during the previous 6 months were negatively associated with condom use compared to those who had not (OR = 0.58, 95% CI = 0.47–0.72). The FSWs more likely to use condoms were those who believed that they were somewhat likely (OR = 1.38, 95% CI = 1.06–1.80) or completely unlikely (OR = 2.37, 95% CI = 1.89–2.96) to contract STIs versus those who believed it was possible that they would contract STIs; those who believed that it was completely unlikely that they might contract HIV versus those who believed it was possible that they might contract HIV (OR = 2.47, 95% CI = 1.93–3.16); those who had more knowledge, as reflected in a score of 5 (OR = 1.70, 95% CI = 1.27–2.27) or 6 (OR = 2.43, 95% CI = 1.94–3.04) on the knowledge scale versus those who had less knowledge, as reflected by a score of 0–4 on this scale; and those who had a score of 1–2 (OR = 3.22, 95% CI = 2.28–4.56) or 3 (OR = 27.72, 95% CI = 21.00–36.60) on the condom-use self-efficacy scale versus those who had a score of 0 on this scale (Table 4).

**Table 4:** Prevention-related correlates of consistent condom use among female sex workers working at hair salons (*n* = 1682)

Variable	Total (N, %)	Consistent condom use (%)	Crude OR	*P* value
Visited a doctor during the previous 6 months				
No	1178 (70.0)	642 (54.5)	1	
Yes	502 (29.8)	206 (41.0)	0.58 (0.47–0.72)	0.000
STI risk perception				
Possible	550 (32.7)	215 (39.1)	1	
Somewhat possible	364 (21.6)	171 (47.0)	1.38 (1.06–1.80)	0.018
Impossible	768 (45.7)	463 (60.3)	2.37 (1.89–2.96)	0.000
HIV risk perception				
Possible	376 (22.4)	139 (37.0)	1	
Somewhat possible	394 (23.4)	170 (43.1)	1.29 (0.97–1.73)	0.081
Impossible	910 (54.1)	538 (59.1)	2.47 (1.93–3.16)	0.000
Knowledge scale				
0–4	503 (29.9)	185 (36.8)	1	
5	302 (18.0)	150 (49.7)	1.70 (1.27–2.27)	0.000
6	873 (51.9)	511 (58.5)	2.43 (1.94–3.04)	0.000
Self-efficacy regarding condom use scale				
0	717 (42.6)	120 (16.7)	1	
1–2	196 (11.7)	77 (39.3)	3.22 (2.28–4.56)	0.000
3	769 (45.7)	652 (84.8)	27.72 (21.00–36.60)	0.000
HIV-related interventions during the previous 6 months				
Received educational material^a^				
No	413 (24.6)	175 (42.4)	1	
Yes	1268 (75.4)	674 (53.1)	1.54 (1.23–1.93)	0.000
Received condoms^a^				
No	443 (26.3)	204 (46.0)	1	
Yes	1239 (73.7)	645 (52.1)	1.27 (1.02–1.58)	0.03
Received face-to-face education from medical staff^a^				
No	852 (50.7)	352 (41.3)	1	
Yes	830 (49.3)	497 (59.9)	2.12 (1.75–2.58)	0.000
Received peer education^a^				
No	1455 (86.5)	708 (48.7)	1	
Yes	227 (13.5)	141 (62.1)	1.73 (1.30–2.31)	0.000
HIV-related interventions scale				
0–1	473 (28.1)	201 (42.5)	1	
2	418 (24.9)	177 (42.3)	0.99 (0.76–1.30)	0.964
3–4	791(47.0)	471 (59.5	1.99 (1.58–2.51)	0.000

^a^This variable was not introduced into the multivariable analysis individually

Those who with a score of 3–4 regarding experience with interventions were more likely to use condoms than those with a score of 0–1 (OR = 1.99, 95% CI = 1.58–2.51). Specifically, those who were exposed to educational materials (OR = 1.54, 95% CI = 1.23–1.93), condom distribution (OR = 1.27, 95% CI = 1.02–1.58), face-to-face education and training from medical staff (OR = 2.12, 95% CI = 1.75–2.58), or peer education (OR = 1.73, 95% CI = 1.30–2.31) were more likely to use condoms than those who were not exposed to the respective intervention.

#### Multivariable analysis

Controlling for possible confounding variables, multivariable analysis revealed that those FSWs who initiated sex work at 20–29 years of age (OR = 0.38, 95% CI = 0.23–0.63) or older than 29 years (OR = 0.39, 95% CI = 0.20–0.79) versus those who initiated sex work before they were 20 years old; those who performed oral sex versus those who did not (OR = 0.54, 95% CI = 0.35–0.82); those who rarely/sometime/often (OR = 0.18, 95% CI = 0.13–0.26) used oral contraceptives versus those who had never used them; and those who had seen a doctor versus those who had not (OR = 0.61, 95% CI = 0.43–0.87) were all negatively associated with condoms use (Table 5). Those who used condoms during their first commercial sex act versus those who did not (OR = 3.83, 95% CI = 2.63–5.58); those who always washed vaginally after commercial sex versus those who never did (OR =2.68, 95% CI = 1.46–4.92); those who used contraception versus those who did not (OR = 1.47, 95% CI = 1.02–2.13); those who had a score of 1–2 (OR = 3.82, 95% CI = 2.44–5.98) or 3 (OR = 26.43, 95%CI = 18.27–38.22) versus a score of 0 on self-efficacy regarding condom use; and those who had a score of 3–4 versus a score of 0–1 regarding their experience of interventions (OR = 2.32, 95% CI = 1.59–3.37) were all more likely to use condoms.

**Table 5:** Multivariate analysis of consistent condom use among female sex workers at hair salons

Variable	Adjusted OR (95% CI)	*P* value
Age of first commercial sex act		
< 20	1	
20–29	0.38 (0.23–0.63)	0.000
> 30	0.39 (0.20–0.79)	0.009
Condom use during first commercial sex act		
No	1	
Yes	3.83 (2.63–5.58)	0.000
Oral sex		
No	1	
Yes	0.54 (0.35–0.82)	0.004
Douche vaginally after commercial sex		
Never	1	
Rarely/sometimes	1.51 (0.78–2.89)	0.219
Often	1.51 (0.79–2.89)	0.211
Always	2.68 (1.46–4.92)	0.002
Oral contraceptive use		
Never	1	
Rarely/sometimes/often	0.18 (0.13–0.26)	0.000
Always	1.26 (0.74–2.13)	0.395
Contraception		
No	1	
Yes	1.47 (1.02–2.13)	0.040
Visited a doctor during the past half year		
No	1	
Yes	0.61 (0.43–0.87)	0.006
Self-efficacy regarding condom use scale		
0	1	
1–2	3.82 (2.44–5.98)	0.000
3	26.43 (18.27–38.22)	0.000
Intervention scale		
0	1	
1–2	1.18 (0.78–1.79)	0.442
3–5	2.32 (1.59–3.37)	0.000

After elimination of the condom-use self-efficacy variable, multivariable analysis showed that those who perceived that it was impossible for them to contract HIV versus those who perceived it was possible (OR = 1.87, 95% CI = 1.36–2.56); those who had a score of 6 for knowledge versus those who had scores of 0–4 for knowledge (OR = 1.67, 95% CI = 1.24–2.26); and those who had 15–29 sexual partners (OR = 1.83, 95% CI = 1.32–2.55) and those who had over 30 sexual partners (OR = 1.52, 95% CI = 1.10–2.09) versus those with fewer than 15 partners appeared to be more likely to use condoms consistently.

## Discussion

To the best of our knowledge, this study is the first to examine the factors associated with condom use among FSWs working at hairs salons in China. Overall, the FSWs in this study were relatively older, had low educational levels, were typically married, had children, and were usually not local residents. Such characteristics are consistent with low-tier FSWs in other parts of China [6, 7, 28, 29]. Lower levels of education may lead to less knowledge about STIs and less exposure to STI prevention and may be associated with less frequent or less proficient condom use; FSWs with lower levels of education who were also older were at greater risk for STI infections [21–23, 30]. The vulnerable demographic characteristics of hair salon FSWs and evident risks of HIV and STIs among low-tier FSWs [17–21]) warrants that hair salon FSWs should be well covered in HIV/STI surveillance and behavioural intervention programs for FSW in China.

We found that the rate of self-reported consistent condom use over the previous month was around 50%, which is lower than the figure reported by recent studies conducted among low-tier FSWs in Xinjiang [22], Guangxi [25] and Guangdong province [23]. Clearly, this rate cannot prevent the spread of HIV and STI within this population. It is notable that the main reason that FSWs did not use condoms was that clients were unwilling to use them. Another key reason was that not using condoms attracted new clients; this implies that, when clients refused to use condoms, some FSWs accepted this arrangement to retain the business and respect the client’s request. Thus, protective sexual behaviour in this group was mainly dependent on the client and not on the FSW herself. Another great concern was that the consistent condom use rate was less than 10% among those FSW who lived together with their spouses or boyfriends. This extremely poor protection could place their regular sexual partners at great risk of HIV/STI infection. Thus, it is essential to raise awareness regarding HIV/STI prevention, to train FSWs in the skills needed to negotiate condom use with clients and regular partners, to enhance the self-efficacy of FSWs regarding safer sex, and to promote HIV prevention intervention programs that include clients and regular partners.

Our study revealed that self-efficacy is the strongest indicator of self-reported consistent condom use. The positive association between condom-use self-efficacy and self-reported consistent condom use has been investigated in other studies among FSWs in China [26, 31]. Additionally, higher scores on self-efficacy were associated with a greater likelihood of condom use among FSWs. These results emphasise the critical importance of promoting self-efficacy education related to condom use among this group of FSWs, as is the case with other FSWs.

The relationships among perceived HIV risk, number of commercial sex partners, HIV-related knowledge, and self-reported consistent condom use did not appear statistically significant in the multiple regression analysis, but these relationships were significant at the bivariable level. However, these correlations lost significance when examined with measures of perceived condom-use self-efficacy, after we eliminated the variable of condom-use self-efficacy in multivariable analysis, those who perceived that it was impossible for them to contract HIV, those who had a higher score of knowledge and those who had more sexual partners were more likely to use condoms consistently. These results are consistent with other studies among low-tier FSWs [22, 24, 25]. We should not ignore the importance of these factors, and future prevention strategies should attach importance to providing information, including about perceived risk and the risk of multiple commercial partners.

Previous research has found that condom use during a person’s first experience with sexual intercourse is associated with condom use in later life [32–34]. We found that condom use during the first commercial sex act was also a predictor of self-reported consistent condom use among FSWs. Behavioural interventions should note that FSWs often do not use condoms during their first commercial sex act and should emphasise the risks of contracting HIV/STI without the use of protection. Those who initiated sex work when they were over the age of 20 were negatively associated with condom use, but the reason for this is unclear. These FSWs might face competition from younger FSWs for clients, may already have given birth and may be using contraception, but not have concerns regarding HIV/STIs. Furthermore, their clients may have a lower socioeconomic status and be less concerned about condom use. These FSWs may also have less power or motivation to use condoms, which may continue to affect their condom use during commercial sex.

Around 21% of FSWs performed oral sex, and we found that those who performed oral sex were negatively associated with condom use than those who did not, and those who performed oral sex were significantly more likely to be younger, to be unmarried, to have earned less money, to have had more sexual partners, to engage in anal sex, and to show STI-related symptoms. Previous research on Chinese samples has indicated that STI clinic attendees [35, 36] and FSWs [37] who performed oral sex were likely to have multiple sexual partners and an increased likelihood of contracting STIs. Those FSWs who performed oral sex were more vulnerable to HIV/STIs. As a result, the HIV/STI intervention programs targeting FSWs should pay particular attention to this subgroup of FSWs.

We found that more frequent vaginal douching was associated with a greater likelihood of using condoms. FSWs who douched may regard this behaviour as a method of protection; therefore, they may be more likely to use both methods to double the protective effects. However, douching may damage normal vaginal flora, which permits the overgrowth of pathogens, and it may also produce a pressurised fluid flow for pathogen transport, which may help to change the location of genital tract infections (which may ascend above the cervix into the uterus, fallopian tubes, or abdominal cavity) [38]. Douching has been associated with higher risks of HIV and HSV-2 infections [17, 30, 39], which suggests that misconceptions about douching should be corrected and education to discourage douching should be provided to this group.

Similarly, those who used contraception were more likely to consistently use condoms, implying that this group of FSWs might have high risk awareness of HIV/STI infections and may also clearly understand that condoms should be used (as contraceptive measures are meant only for contraception). Interestingly, rarely/sometimes/often using oral contraceptives were indicative of inconsistent or non-use of condoms. Thus, this group of FSWs maybe not aware of their risk of pregnancy and HIV/STI infection and, therefore, use neither oral contraceptives nor condoms. It is necessary to educate FSWs to use condoms to prevent unwanted pregnancies and HIV/STI transmission.

Our study confirmed that those who used condoms inconsistently were more vulnerable to STI infection and were, therefore, more likely to contract STIs and visit a doctor for diagnosis and treatment. This finding suggests that doctors at STI clinics should be encouraged to provide safer sex education while offering screening and treatment for STIs.

Previous studies among low-tier FSWs showed that peer education [23, 25], free condoms and educational materials [24], and behavioural interventions [23] were protective factors leading to condom use. FSWs who were exposed to 3–4 types of intervention were likely to consistently use condoms, but there was no association in this regard among those who have experienced no or one type of intervention. The behavioural interventions offered to this group of FSWs should include different components and should be delivered with some degree of intensity to promote the consistent use of condoms.

We should interpret these results with caution due to the limitations of the study. First, our findings may not be generalisable to other areas of China due to the huge size of China and the diversity of low-tier FSWs. Additionally, it is very difficult to estimate the exact number of low-tier FSWs working at various venues because sex work is illegal and highly stigmatised. Furthermore, as sexual behaviour is a sensitive subject in China, we did not know what proportion of low-tier FSWs participated in the survey and could not determine differences in the characteristics between those who participated and those who did not. In addition, we could not ascertain differences in the characteristics between those who worked in the counties which implemented AIDS Care project, and those who did not. Because our study design is cross-sectional, the associations between condom use and various factors cannot be interpreted as reflecting causality. Thus, these relationships warrant further research using a prospective design. Finally, our data were subject to biases of social desirability and self-selection, especially self-reports of sexual behaviours.

## Conclusion

Although this study has limitations, it also has several strengths. Our study was conducted in various areas of all 11 prefectures within Zhejiang province, and the research subjects were all low-tier FSWs in various venues in these counties. Our findings confirm that FSWs working at hair salons, similar to other low-tier FSWs, are at risk of contracting and/or transmitting HIV/STI, as sexual encounters with their clients are not adequately protected. Our findings suggest that condom use during the first commercial sex act, douching vaginally after commercial sex, having ever used contraception, perceived self-efficacy regarding condom use, and having ever been exposed to interventions are protective factors of consistent condom use. Of these, perceived self-efficacy was the strongest predictor of condom use, whereas initiation of commercial sex at a later age, performing oral sex, rarely/sometimes/often using contraceptives, and having seen a doctor during the past 6 months were the strongest predictors of not using condoms. Future intervention activities directed toward FSWs in China need to be tailored to the particular target group and should consider the characteristics of FSWs to ensure that these programs are responsive to the needs of their recipients and effective.

## Abbreviations

FSW: Female sex worker
STI: Sexually transmitted infection

## References

[1] Baral S, Beyrer C, Muessig K, Poteat T, Wirtz AL, Decker MR, Sherman SG, Kerrigan D (2012). Burden of HIV among female sex workers in low-income and middle-income countries: a systematic review and meta-analysis. Lancet Infect Dis.

[2] Prüss-Ustün A, Wolf J, Driscoll T, Degenhardt L, Neira M, Calleja JM (2013). HIV due to female sex work: regional and global estimates. PLoS One.

[3] Tucker JD, Yin YP, Wang B, Chen XS, Cohen MS (2011). An expanding syphilis epidemic in China: epidemiology, behavioural risk and control strategies with a focus on low-tier female sex workers and men who have sex with men. Sex Transm Infect.

[4] Dalla RL, Baker LM, DeFrain J, Williamson C (2013). Global perspectives on prostitution and sex trafficking: Africa, Asia, Middle East, and Oceania.

[5] Zhang C, Li X, Hong Y, Zhou Y, Liu W, Stanton B (2013). Unprotected sex with their clients among low-paying female sex workers in southwest China. AIDS Care.

[6] Li J, Chen XS, Merli MG, Weir SS, Henderson GE (2012). Systematic differences in risk behaviors and syphilis prevalence across types of female sex workers: a preliminary study in Liuzhou, China. Sex Transm Dis.

[7] Chang H, Zhi X, Chen X-S, Cohen MS. Systematic review and meta-analysis of syphilis seroprevalence among female sex workers in China: Bethesda: NIH Fogarty International Clinical Scholar Conference; 2010.

[8] Hong Y, Zhang C, Li X, Fang X, Lin X, Zhou Y, Liu W (2012). HIV testing behaviors among female sex workers in Southwest China. AIDS Behave.

[9] Wang Q, Yang P, Gong XD, Jiang J, Yang B (2009). Syphilis prevalence and high risk behaviors among female sex workers in different settings. China J AIDS STD.

[10] Yang X, Xia G (2006). Gender, work, and HIV risk: determinants of risky sexual behavior among female entertainment workers in China. AIDS Educ Prev.

[11] Yang X, Xia G, Li X, Latkin C, Celentano D (2010). Social influence and individual risk factors of HIV unsafe sex among female entertainment workers in China. AIDS Educ Prev.

[12] Fornasa CV, Gai F, Tarantello M, Gallina P (2005). Knowledge of sexually transmitted diseases and condom use among female street sex workers in Padua. Acta Dermatovenerol Alp Pannonica Adriat.

[13] Mondal NI, Hossain K, Islam R, Mian AB (2008). Sexual behavior and sexually transmitted diseases in street-based female sex workers in Rajshahi City, Bangladesh. Braz J Infect Dis.

[14] Huang Y, Maman S, Pan S (2012). Understanding the diversity of male clients of sex workers in China and the implications for HIV prevention programmes. Glob Public Health.

[15] Zhou Y, Li X, Zhang C, Tan G, Stanton B, Zhang X, Cui Y (2013). Rates of HIV, syphilis, and HCV infections among different demographic groups of female sex workers in Guangxi China: evidence from 2010 national sentinel surveillance data. AIDS Care.

[16] Uretsky E (2008). 'Mobile men with money': the socio-cultural and politico-economic context of 'high-risk' behaviour among wealthy businessmen and government officials in urban China. Cult Health Sex.

[17] Wang H, Chen RY, Ding G (2009). Prevalence and predictors of HIV infection among female sex workers in Kaiyuan City, Yunnan Province, China. Int J Infect Dis.

[18] Rahman M, Alam A, Nessa K, Hossain A, Nahar S, Datta D, Alam Khan S, Amin Mian R, Albert MJ (2000). Etiology of sexually transmitted infections among street-based female sex workers in Dhaka, Bangladesh. J Clin Microbiol.

[19] Wang H, Wang N, Bi A, Wang G, Ding G, Jia M, Lu L, Smith K (2009). Application of cumulative odds logistic model on risk factors analysis for sexually transmitted infections among female sex workers in Kaiyuan city, Yunnan province, China. Sex Transm Infect.

[20] Zhang L, Chow EP, Su S, Yiu WL, Zhang X, Iu KI, Tung K, Zhao R, Sun P, Sun X, Yuan L, Muessig KE, Tucker JD, Jing J (2015). A systematic review and meta-analysis of the prevalence, trends, and geographical distribution of HIV among Chinese female sex workers (2000-2011): implications for preventing sexually transmitted HIV. Int J Infect Dis.

[21] Chen XS, Wang QQ, Yin YP, Liang GJ, Jiang N, Yang LG, Liu Q, Zhou YJ, Huan XP, Wei WH, Wang B (2012). Prevalence of syphilis infection in different tiers of female sex workers in China: implications for surveillance and interventions. BMC Infect Dis.

[22] Ni M, Guo N, Hu X, Chen X, Hu X, Ma Y, Wang S (2015). Analysis on influencing factors of unprotected commercial sexual behavior among low-grade female sex workers. Chin health care women child.

[23] Wang L, Wen M, Li W, Yan J, Li D, Liang Y, Lin Y, Zhang D, Zhou L (2015). Knowledge awareness, condom use and affecting factors related to HIV/AIDS in low-tier female sex workers in Jiang men. Chin J AIDS/STDS..

[24] Bo J, Zhou N, Dong X, Chen C (2010). Condom use among commercial sex workers in low class establishments and related factors. Chin J AIDS/STDS.

[25] Li S, Lin X, Xu Y, Huang C, Lin Q, Li Q, Zhang S, Wei X (2014). Analysis on HIV/STD infection, condom use and its determinants among low-grade female sex workers in rural areas. Mod prev med.

[26] Zhao J, Song F, Ren S (2012). Predictors of condom use behaviors based on the health belief model (HBM) among female sex workers: a cross-sectional study in Hubei Province, China. PLoS One.

[27] Zhejiang provincial bureau of statistics. The provincial conditions of Zhejiang. Available at: http://tjj.zj.gov.cn/zjsq1/zrdl/. Accessed 28 November 2016.

[28] Hong Y, Li X (2008). Behavioral studies of female sex workers in China: a literature review and recommendation for future research. AIDS Behav.

[29] Yi H, Mantell JE, Wu R, Lu Z, Zeng J, Wan Y (2010). A profile of HIV risk factors in the context of sex work environments among migrant female sex workers in Beijing, China. Psychol Health Med.

[30] Wang H, Wang N, Chen RY, Sharp GB, Ma Y, Wang G, Ding G, Wu Z (2008). Prevalence and predictors of herpes simplex virus type 2 infection among female sex workers in Yunnan Province, China. Int J STD AID.

[31] Zhang L, Li X, Zhou Y, Lin D, Su S, Zhang C, Stanton B (2015). Predictors of consistent condom use among Chinese female sex workers: an application of the protection motivation theory. Health Care Women Int.

[32] Ma Q, Ono-Kihara M, Cong L, Pan X, Xu G, Zamani S, Ravari SM, Kihara M (2009). Behavioral and psychosocial predictors of condom use among university students in Eastern China. AIDS Care.

[33] Shafii T, Stovel K, Davis R, Holmes K (2004). Is condom use habit forming?: Condom use at sexual debut and subsequent condom use. Sex Transm Dis.

[34] Klavs I, Rodrigues LC, Wellings K, Weiss HA, Hayes R (2005). Increased condom use at sexual debut in the general population of Slovenia and association with subsequent condom use. AIDS.

[35] Ke Y. Survey on oral sexual activity among STI patients. Chin J Derm Venereal. 2001;15(4):201–1.

[36] Ma Q, Pan X, Cai G, Yan J, Xu Y, Ono-Kihara M, Kihara M (2013). The characteristics of heterosexual STD clinic attendees who practice oral sex in Zhejiang Province, China. PLoS One.

[37] Zhi H, Xu L, Hou L, Ding R (2001). Investigation of the oral sexual behaviors conducted by sex illegality woman. World J Infect.

[38] Zhang J, Thomas AG, Leybovich E (1997). Vaginal douching and adverse health effects: a meta-analysis. Am J Public Health.

[39] Luo L, Xu JJ, Wang GX, Ding GW, Wang N, Wang HB (2016). Vaginal douching and association with sexually transmitted infections among female sex workers in a prefecture of Yunnan Province, China. Int J STD AIDS.

